# Emergence of Clonally-Related South Asian Clade I Clinical Isolates of *Candida auris* in a Greek COVID-19 Intensive Care Unit

**DOI:** 10.3390/jof9020243

**Published:** 2023-02-11

**Authors:** Maria Katsiari, Angeliki Mavroidi, Nikolaos Kesesidis, Eleftheria Palla, Konstantina Zourla, Kyriakos Ntorlis, Konstantinos Konstantinidis, Maria Laskou, Konstantinos Strigklis, Anastasios Sakkalis, Charikleia Nikolaou, Evangelia D. Platsouka, Ioannis Karakasiliotis, Georgia Vrioni, Athanasios Tsakris

**Affiliations:** 1 Intensive Care Unit, Konstantopouleio-Patission General Hospital, 14233 Athens, Greece; 2 Department of Microbiology, Konstantopouleio-Patission General Hospital, 14233 Athens, Greece; 3 Laboratory of Biology, Department of Medicine, Democritus University of Thrace, 68100 Alexandroupolis, Greece; 4 Department of Microbiology, Medical School, National and Kapodistrian University of Athens, 75 MikrasAsias Street, 11527 Athens, Greece

**Keywords:** *Candida auris*, intensive care unit, COVID-19, antifungal resistance, sequencing

## Abstract

*Candida auris* has recently emerged as a multidrug-resistant yeast implicated in various healthcare-associated invasive infections and hospital outbreaks. In the current study, we report the first five intensive care unit (ICU) cases affected by *C. auris* isolates in Greece, during October 2020–January 2022. The ICU of the hospital was converted to a COVID-19 unit on 25 February 2021, during the third wave of COVID-19 in Greece. Identification of the isolates was confirmed by Matrix Assisted Laser Desorption Ionization Time of Flight mass spectroscopy (MALDI-TOF]. Antifungal susceptibility testing was performed by the EUCAST broth microdilution method. Based on the tentative CDC MIC breakpoints, all five *C. auris* isolates were resistant to fluconazole (≥32 μg/mL), while three of them exhibited resistance to amphotericin B (≥2 μg/mL). The environmental screening also revealed the dissemination of *C. auris* in the ICU. Molecular characterization of *C. auris* clinical and environmental isolates was performed by MultiLocus Sequence Typing (MLST) of a set of four genetic loci, namely ITS, D1/D2, RPB1 and RPB2, encoding for the internal transcribed spacer region (ITS) of the ribosomal subunit, the large ribosomal subunit region and the RNA polymerase II largest subunit, respectively. MLST analysis showed that all isolates possessed identical sequences in the four genetic loci and clustered with the South Asian clade I strains. Additionally, PCR amplification and sequencing of the CJJ09_001802 genetic locus, encoding for the “nucleolar protein 58” that contains clade-specific repeats was performed. Sanger sequence analysis of the TCCTTCTTC repeats within CJJ09_001802 locus also assigned the *C. auris* isolates to the South Asian clade I. Our study confirms that *C. auris* is an emerging yeast pathogen in our region, especially in the setting of the ongoing COVID-19 worldwide pandemic. Adherence to strict infection control is needed to restrain further spread of the pathogen.

## 1. Introduction

Candidemia and other forms of invasive candidiasis, including infections of normally sterile body fluids, deep tissues and organs, are associated with prolonged hospitalization, increased health care costs and poor outcomes [1,2]. Although *Candida albicans* remains the most often isolated species, a shift towards *Candida* non-*albicans* species has been observed recently, which is thought to be driven mainly by the increasing use of prophylactic antifungal agents [3]. *Candida auris* is an emerging multidrug-resistant yeast that has been implicated in healthcare-associated outbreaks and various invasive infections. It was first reported in 2009, after being isolated from the external ear canal discharge of a patient in Japan [4]. However, *C. auris* was also found incidentally by molecular identification of unidentified yeasts, which were recovered in 1996 as bloodstream isolates [5]. Thereafter, cases of persistent colonization and invasive infections have been described in more than 40 countries located on six continents [6,7]. At the present time, *C. auris* is divided into four major clades displaying distinct biological and drug resistance patterns: I, Southern Asian; II, Eastern Asian; III, South African; IV, South American. Recently, clade V has been identified in Iran showing a difference of >200,000 single-nucleotide polymorphisms (SNPs) from the other clades [8].

There are several reasons for *C. auris* being a serious concern for public health. Firstly, identification of *C. auris* remains challenging since most phenotypic methods misidentify it. *C. auris* typically forms smooth, shiny, pink or whitish-gray, viscous colonies on growth media, while it has high tolerance for salinity and heat [9,10,11,12]. Nevertheless, other *Candida* species, such as *C. haemulonii*, *C. duobushaemulonii* and *C. lusitaniae* are closely related and share similar phenotypic characteristics. The most common diagnostic platforms available in clinical and public health laboratories (e.g., VITEK2) often misidentify *C. auris* [10,11,12,13]. Accurate identification methods for *C. auris* include matrix-assed laser desorption ionization-time of flight (MALDI-TOF) platforms, polymerase chain reaction, sequencing, and amplified fragment length polymorphism fingerprinting.

Moreover, *C. auris* exhibits a high propensity to contaminate healthcare environments, resulting in clonal transmission. Among its characteristics are the thermo- and halotolerance, along with inability to flourish in anaerobic and acidic conditions [14]. Patients shed *C. auris* in their close environment. *C. auris* contamination has been found on bed rails, bed pans, mattresses, linen, pillows, furniture, door handles, flooring, walls, radiators and windowsills, bathing areas, sinks and cleaning equipment [6,7]. Moreover, medical equipment has also been found contaminated through contact with patients. Notably, *C. auris* may survive for seven days on steel and porous surfaces and for 14 days on plastics, while it can further survive in anon-culturable state on plastic surfaces for up to four weeks [15,16]. Lastly, *C. auris* exhibits resistance to fluconazole and variable susceptibility to other azoles, amphotericin B, and echinocandins. *C. auris* isolates are often multidrug-resistant and there are several reports of resistant isolates to triazoles, presumably due to their abuse in marginalized populations, resistant strains to polyenes depending on the region where the strain is isolated, and echinocandin-resistant strains in India and South Africa. Although high levels of intrinsic (e.g., polyenes) and acquired resistance (e.g., azoles and echinocandins) of Candida spp. have been well documented, the knowledge of the molecular resistance mechanisms of *C. auris* is still unclear [17,18]. *C. auris’* ability to resist to antifungal drugs, along with the inability of commonly used disinfectants in healthcare settings to eradicate *C. auris*, further complicate the problem.

In Greece, the first report of *C. auris* in 2019 involved a sporadic case of male cystic fibrosis patient in his 20s, who presented with respiratory exacerbation [19]. In the current study, we report for the first time, the dissemination of South Asian clade I *C. auris* isolates among patients hospitalized during a 15 month-period of the COVID-19 pandemic in a Greek multidisciplinary intensive care unit (ICU). According to our knowledge, molecular characterization of *C. auris* outbreaks has not been previously performed in Greece.

## 2. Materials and Methods

### 2.1. Setting, Definition of Cases, Demographic and Clinical Characteristics of C. auris Cases

“Konstantopouleio-Patission” is a tertiary care hospital in Athens, Greece (with approximately three million inhabitants), with a maximum of 280 beds in total, including: a nine-bed multidisciplinary ICU, internal medicine, cardiology, surgical, urology and other wards. The study was conducted during October 2020–January 2022 and a case was defined as any patient hospitalized in the ICU and infected with *C. auris*, during routine surveillance of cultures from various clinical specimens of ICU patients. The ICU of the hospital was converted to a COVID-19 unit on 25 February 2021, during the third wave of COVID-19 in Greece. Patient 1 was non-COVID-19, whereas patients 2–5 were admitted in the ICU as COVID-19 positive. It should be mentioned that all COVID-19 patients had received corticosteroid therapy with dexamethasone; patient 5 had additionally received tocilizumab. The demographic and clinical characteristics of the five ICU patients infected with *C. auris* are presented in Table 1.

The first patient (patient 1) was a 42-year-old man with traumatic paraplegia and diabetes mellitus, who was admitted in hospital with a6-day fever and shortly was intubated due to septic shock and lactic acidosis (Table 1). Upon ICU admission, he presented with acute respiratory distress syndrome (ARDS), severe hemodynamic instability and oliguria. He was treated for presumable urosepsis with broad spectrum antibiotics, including fluconazole, and his condition was ameliorated. On day 22, he presented new septic shock and multiple cultures were obtained. *C. auris,* along with other multidrug resistant pathogens (*Acinetobacter baumannii*, *Klebsiella pneumoniae*, *Enterococcus faecium*) from a deep hip decubitus ulcer were isolated. Concurrently, *C. auris* was isolated from a urine culture. The patient was then administrated with micafungin. Despite treatment, the same pathogens, including *C. auris*, were isolated from the hip decubitus ulcer on day 44. The patient expired on day 49, due to septic shock and multi-organ failure. Of note, no other cultures from contemporary patients, or environmental samples revealed *C. auris*.

Patient 2 was a 57-year-old woman with metastatic adenocarcinoma of rectum, who had undergone a nephrostomy and a ureterostomy. She was transferred to our ICU from the surgical ward of another hospital due to septic shock, lactic acidosis and acute renal failure. Upon admission, blood and urine cultures disclosed *C. albicans* and vancomycin-resistant *E. faecium*, while on day 8 multidrug resistant *A. baumannii* was isolated from blood cultures. On day 9, *C. auris* was isolated from a urine sample obtained from ureterostomy. The patient was successfully treated with meropenem, daptomycin and fluconazole and transferred to a surgical ward on day 10.

Patient 3 was a 72-year-old woman with diabetes mellitus, arterial hypertension and asthma. On day 13, *C. auris* was isolated from a surveillance urine culture. She received a 14-day course with caspofungin without attainment of urine sterilization. Notably, on day 28, the patient suffered from catheter-related bloodstream infection due to multidrug resistant *A. baumannii*, which was successfully treated with central line removal and administration of meropenem plus colistin. The patient was hospitalized in the ICU for 44 days and afterwards transferred to a rehabilitation center.

Patient 4 was a 78-year-old man with diabetes mellitus, arterial hypertension and a recently placed stent in the common bile duct due to painless jaundice. Upon admission, blood cultures disclosed vancomycin-resistant *E. faecium*. On day 9, *C. auris* and *C. parapsilosis* were isolated from blood samples and *C. auris* also in urine; thus, he was started a course of caspofungin. On day 20, amphotericin B was added due to persistence of leukopenia and mild septic condition. A computed tomography was performed and was not indicative of stent contamination. *C. auris* isolation in urine cultures persisted until his death (day 37).

The last patient (patient 5) was a 64-year-old woman. Her medical record included rheumatoid arthritis under treatment with prednizolone, renal resection due to adenocarcinoma for the past six years, and Hashimoto thyroiditis. On day 12, she developed septic shock due to *E. faecium* and *A. baumannii* and the antimicrobial regimen was escalated to meropenem, tigecycline and daptomycin. On day 14, *C. auris* was isolated from bronchial secretions, while being on itraconazole. The patient expired due to septic shock on day 20.

### 2.2. Culture, Identification of the Isolates, Antimicrobial Susceptibility Testing and Environmental Screening

Bacterial isolates and yeasts from various clinical specimens were identified and antimicrobial susceptibility testing was performed by the Vitek2 Compact15 automated system (Biomerieux, Paris, France) ona routine basis at the hospital clinical laboratory. Yeasts were grown on Sabouraud Dextrose agar at 35 °C and 42 °C. Additionally, CHROMagar^TM^ Candida Plus agar was used and pale cream colonies with a distinctive blue halo, suspected as *C. auris*, were sent to the Department of Microbiology, Medical School, National and Kapodistrian University of Athens, Greece for further analysis. Identification of the isolates was confirmed by MALDI-TOF spectrometry, which is one of the most efficient diagnostic techniques for accurate identification of *C*. *auris* using the Microflex LT (Bruker Daltonics, Bremen, Germany) platform. Susceptibility to antifungal agents was evaluated by the European Committee on Antimicrobial Susceptibility Testing (EUCAST) standardized broth microdilution method [20] and interpretation of minimum inhibitory concentration (MIC) values was based on U.S.A. Centers for Disease Control and Prevention (CDC) proposed breakpoints [21].

The Infectious Disease Control Committee of the hospital was informed, and environmental screening was performed, when three patients (patients 2–4) were concurrently hospitalized in the ICU during August–September 2021. Cultures from three environmental samples (surface samples from beds and side tables) during this period have also yielded this pathogen.

### 2.3. Sequencing and Phylogenetic Analysis of C. auris Isolates

PCR amplification and sequencing of four housekeeping genetic loci of the MLST scheme used for molecular typing of *C. auris* were performed based on previous studies, including the internal transcribed spacer (ITS) of the ribosomal DNA (rDNA), the D1/D2 large ribosomal subunit regions, as well as RPB1 and RPB2 of the RNA polymerase II largest subunit [22,23,24]. After DNA extraction from cultivated *C. auris* strains from clinical and environmental samples on Sabouraud dextrose agar, the ITS region of the ribosomal subunit was amplified using the ITS-1 (5′-TCCGTAGGTGAACCTTGCGG-3′) and ITS-4 (5′-TCCTCCGCTTATTGATATGC-3′) set of primers, while the D1/D2 region was amplified using primers NL-1 (5′-GCATATCAATAAGCGGAGGAAAAG-3′) and NL-4 (5′-GGTCCGTGTTTCAAGACGG-3′). The RPB1 gene was amplified using the RPB1af (5′-GARTGYCCDGGDCAYTTYGG-3′) and RPB1cr (5′-CCNGCDATNTCRTTRTCCATRTA-3′) primers and amplification of RPB2 was performed using primers RPB2-5F (5′-GAYGAYMGWGATCAYTTYGG-3′) and RPB2-7Cr (5′-CCCATRGCTTGYTTRCCCAT-3′). All sequencing reactions were carried out using the respective forementioned primer sets. Specific genetic loci containing short tandem repeat (STR) markers can also be used to differentiate *C. auris* clades. PCR amplification and sequencing was performed for one of these markers, using the M9a primer pair [24]. All nucleotide sequences obtained after the PCR amplification and Sanger sequencing of each examined genetic locus were aligned by MUSCLE [25] in order to investigate the origin, clade classification and possible divergence between the studied *C. auris* isolates. *C. auris* strains from both clinical and environmental samples possessed identical sequences in the examined genetic loci.

Nucleotide ITS, D1/D2, RPB1 and RPB2 sequences of various *C. auris* strains were concatenated bioinformatically [26] and also included for the construction of the phylogenetic trees, as representatives of known *C. auris* clades according to the literature, obtained from the National Center for Biotechnology Information (NCBI) website (https://www.ncbi.nlm.nih.gov/), as follows: B11205 (CP060353-CP060359), B13916 (CP060374-CP060380), B11220 (CP043531-CP043537), B12043 (CP050666-CP050672), B11809 (CP050659-CP050665), B13463 (CP050652-CP050658), B12037 (CP060367-CP060373), B12631 (CP060360-CP060366), B17721 (CP060353-CP060359), B11245 (CP043442-CP043448), B12342 (CP060346-CP060352), and B18474 (CP050673-CP050679), BJCA001 (CP068451-CP068458), CA1LBN (CP077052-CP07705). A total of 15 nucleotide sequences and 2713 positions in the final dataset were included. All nucleotide sequences were then refined through default MUSCLE alignment, as provided by the MEGA11 software [27]. Lastly, the phylogenetic reconstruction was performed using the neighbor joining (NJ) substitution model. The bootstrap consensus tree inferred from 500 replicates was taken to represent the evolutionary history of the taxa analyzed. The evolutionary distances were computed using the Kimura 2-parameter method and are in the units of the number of base substitutions per site. Codon positions included were 1st + 2nd + 3rd + Noncoding. All ambiguous positions were removed for each sequence pair (pairwise deletion option).

## 3. Results

### 3.1. Timeline of the Five C. auris Cases in the ICU

**Figure 1:**
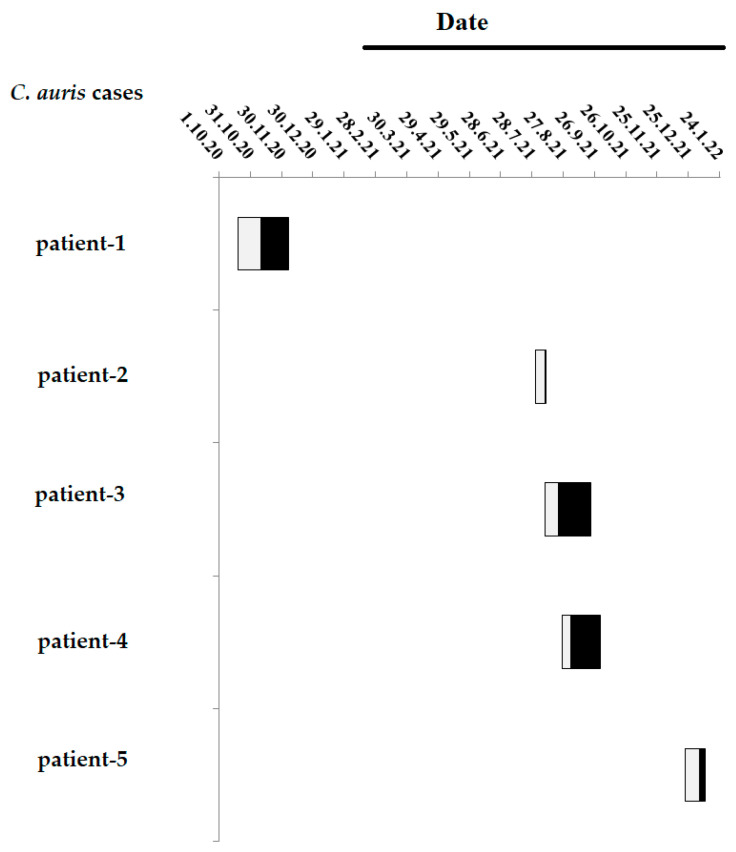
Timeline of the five *C. auris* cases in the ICU. The boxes denote the length of stay (LOS) of *C. auris* cases in the ICU prior (grey boxes) and post to (black boxes) the isolation of *C. auris*. The black line denotes the period that the ICU has been functioned as a COVID-19 ICU.

The timeline of the five *C. auris* cases in the ICU is presented in Figure 1. The first patient (patient 1) was admitted in the ICU on October 2020, when the ICU was non-COVID-19. As mentioned earlier, the ICU of the hospital was converted into a COVID-19 unit on 25 February 2021. The next four patients (patients 2 to 5) were hospitalized in the ICU during August 2021–January 2022, when the ICU was COVID-19. Patients 2 to 4 were hospitalized concurrently in the ICU during August–September 2021. The last patient (patient 5) was hospitalized on January 2022, four months after the aforementioned ICU dissemination.

### 3.2. Antifungal Susceptibility Testing

The antifungal susceptibility testing of *C. auris* isolates recovered from the five ICU patients is presented in Table 2. Based on the tentative CDC MIC breakpoints for fluconazole (≥32 μg/mL), amphotericin B (≥2 μg/mL), micafungin (<4 μg/mL) and anidulafungin (<4 μg/mL), all isolates were resistant to fluconazole (MICs > 128), while three of them exhibited resistance to amphotericin B (MICs ≥ 2 μg/mL). All isolates were susceptible to micafungin (MICs 0.12–0.5) and anidulafungin (MICs 0.12–0.5).

### 3.3. Molecular Typing

**Figure 2:**
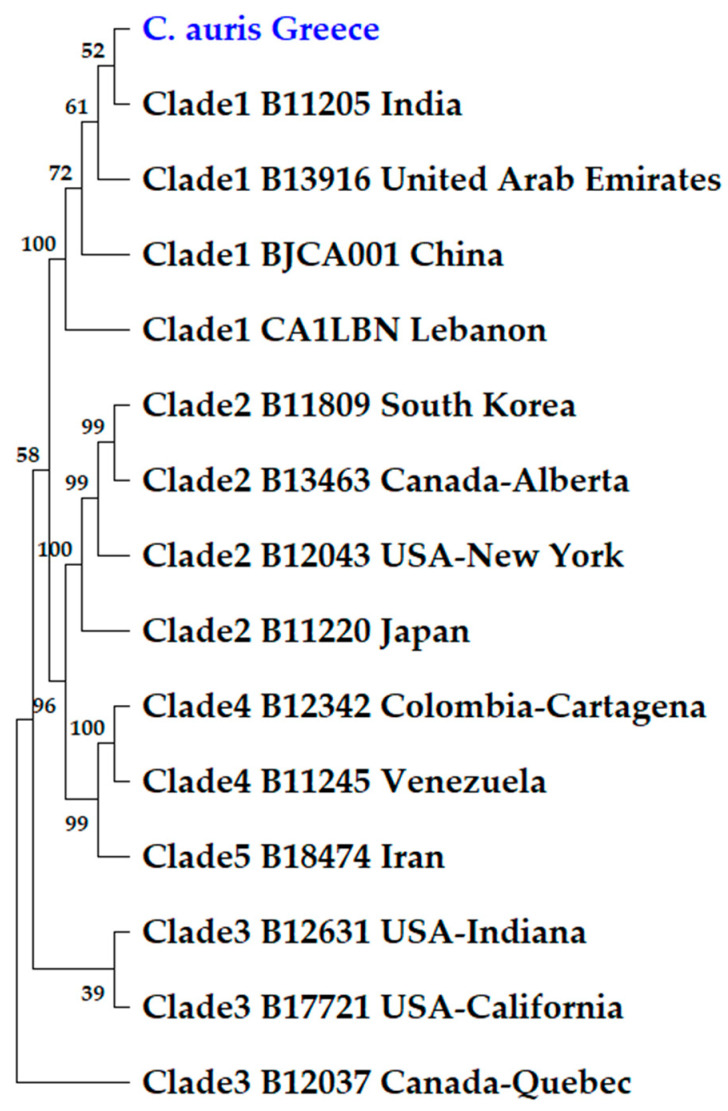
Evolutionary relationships based on the concatenated ITS, RPB1, RPB2 and D1/D2-nucleotide sequences of the *C. auris* isolates identified in this study and other strains belonging to clades Ι to V. The identifier *C. auris* strain names and countries of isolation are depicted. Branches corresponding to partitions reproduced in less than 50% bootstrap replicates were collapsed. The percentage of replicate trees in which the associated taxa clustered together in the bootstrap test (500 replicates) are shown next to the branches.

The ITS, D1/D2, RBP1 and RPB2 genetic loci are often used for the molecular typing of *Candida* species, while also showing intra-species variation [22]. Sanger sequence analysis for these four genomic regions was used to investigate the phylogenetic relationships of the studied *C. auris* isolates and identify the cluster/clade in which they belonged. However, prior to molecular typing and phylogenetic analysis of the studied *C. auris* isolates, the respective ITS, D1/D2, RPB1 and RPB2 sequenced regions were aligned by MUSCLE and each output nucleotide sequence alignment demonstrated that all of the studied *C. auris* isolates (both clinical and environmental) possessed identical sequences. Phylogenetic analysis was performed subsequently based on the ITS, D1/D2, RPB1 and RPB2 nucleotide sequences of the isolates, which were bioinformatically fused (concatenated), as stated. The studied *C. auris* isolates phylogenetically clustered within clade I with a bootstrap value of 100 and were closely related with the B11205 *C. auris* strain from India with a bootstrap value of 52 (Figure 2). Sanger sequence analysis of the TCCTTCTTC repeats within CJJ09_001802 locus, encoding the “nucleolar protein 58” that contains clade-specific repeats, also demarcated the strain with clade I (18 repeats, clade I, Supplementary Figure S1). As the CJJ09_001802 region lacked differentiating mutations apart from the number of TCCTTCTTC repeats, the analysis could not observe phylogenetic relationships with other *C. auris* epidemic strains.

## 4. Discussion

In the present study, we reported a case-series of ICU patients with *C. auris*, who were hospitalized in a nine-bed Greek ICU during different time points. The first case occurred before ICU’s operation as ICU-COVID-19 and represented a single case, since no other contemporary patient, nor the environmental samples, revealed *C. auris*. The next three cases (patients 2–4) clustered within a 25-day period, during the fourth wave of COVID-19 occurred in Greece, when the ICU was operated as a COVID-19 ICU. The environmental screening also revealed *C. auris* isolates with identical sequences in the examined genetic loci, suggesting its horizontal transmission. Of note, patient 2 has been transferred in the ICU from another hospital of Athens, Greece and, according to our knowledge, this hospital was surveyed for potential *C. auris* contamination. The last case appeared four months later. According to MLST, all five *C. auris* clinical isolates belonged to clade I (clustered with South Asian strains), as the first reported case from our country [19], suggesting that this pathogen is circulating in Greece at least as of 2019. In more detail, the phylogenetic analysis based on the concatenated ITS, RPB1, RPB2 and D1/D2-nucleotide sequences of the *C. auris* isolates identified in the current survey and other isolates belonging to clades Ι to V has shown that all five clade I *C. auris* of our collection were closely related to those described from India. Moreover, antifungal susceptibility and sequence analysis results of *C. auris* isolates of the present study were in accordance with previous studies, as clade I has shown the greatest percentage of isolates resistant to fluconazole (97%) and amphotericin B (47%) [22].

Increasing reports of *C. auris* outbreaks among COVID-19 patients have been published recently [28,29,30,31]. Similarly to our cases, COVID-19 patients are usually hospitalized in ICUs for prolonged periods and receive mechanical ventilation and multiple courses of broad-spectrum antibiotics. Immune deregulation associated with COVID-19 along with treatment with anti-interleukin-6 (anti-IL-6) agents, such as tocilizumab and corticosteroids, make these patients susceptible to fungal infections [32,33]. Moreover, *C. auris* demonstrates high propensity to develop antifungal resistance under selective pressure [18]. Recent research has shown that most *C. auris* strains show higher resistance to fluconazole, followed by amphotericin B, and less resistance to 5-fluorocytosine and caspofungin [7,18,34,35,36,37]. Echinocandins are recommended as the first line empiric therapy for *C. auris* infections, pending susceptibility testing. In order to provide better efficacy due to synergistic interactions, combined treatment with echinocandin and liposomal amphotericin B has been prescribed in unresponsive cases to echinocandins, as in patient 1 with the soft tissue infection.

*C. auris* has been implicated in various infections, such as bloodstream and urinary tract infections, otitis, skin and bone infections, myocarditis and meningitis [7]. However, isolation of *C. auris* from non-sterile body sites, such as skin, lungs or urinary and genital tract, may not always reflect infection. Indeed, we can speculate that *C. auris* in patients 2, 3 and 5 rather represented colonization, since signs or symptoms of infection were not obvious at isolation time. In any case, it is crucial to identify *C. auris* even from a non-sterile body site because of the risk of contact transmission. Unlike other *Candida* species, *C. auris* features a propensity to colonize certain superficial body sites, such as the groin, axilla and nares, but not the intestinal tract [8,14]. On the other hand, patients who have received treatment in healthcare facilities can be persistently colonized (nares, groin, axilla, skin, urinary tract, vagina and rectum) from 1 month to 3 years, and perhaps indefinitely [8]. Similarly, in all our patients whose subsequent cultures were tested (patients 1, 3 and 4). *C. auris* isolation persisted until their discharge from the ICU. A substantial defense mechanism that protects *C. auris* against antifungals is biofilm formation. *C. auris* isolates are able to form either low or high biomass biofilms depending on the micro-environment, which can be highly resistant to all classes of antifungals through multiple mechanisms [34,35,36,37]. Thus, cases resistant to all available antifungals have been reported. There is currently no known decolonization strategy. Environmental cleaning is fundamental for preventing horizontal transmission within a healthcare facility. Proper disinfection of reusable medical equipment is also mandatory. Furthermore, screening should be applied for patients previously hospitalized in a healthcare environment with confirmed *C. auris* isolation.

However, the most alarming issue is the *C. auris* transmission within the ICU environment. During the COVID-19 pandemic, extended use of the underlayer protective equipment, multiple gowns and glove layers, lapses in cleaning and disinfection procedures, deficient adherence to hand hygiene by the health care personnel, along with low nurse to patient ratio, may contribute to widespread transmission of *C. auris.* Epidemiological investigations may elucidate the modes of *C. auris* nosocomial transmission. Previous studies by using MLST have shown that that *C. auris* strains are highly related despite being isolated from patients admitted to a large number of hospitals, suggesting that clonal strains are circulating or that the method is insufficiently discriminatory [38]. On the other hand, a microsatellite system for a largely clonal pathogen is a labor-intensive process. Whole Genome Sequence (WGS) has a high discriminatory power that may elucidate hospital transmission, although it is a costlier approach. In *C. auris*, whole Genome Sequence (WGS) analysis has revealed nearly simultaneous, recent and independent emergence of different clonal populations on three continents [39,40]. Several studies have utilized single nucleotide polymorphisms (SNPs), short tandem repeat, and MLST strategies for molecular typing of *C. auris* outbreaks. However, a standardized dataset to allow for comparisons of these molecular typing and phylogenomic pipelines has not been established for *C. auris*. Recently, a genomic benchmark dataset has been developed to serve as a resource to facilitate global efforts to collaborate and rapidly validate sequence analysis tools, which should be able to differentiate between the geographic clades, hospital transmission, colonization and the extent of carriage in the community [41,42].

In the present study, by using an MLST analysis and a single STR marker, we have shown that *C. auris* isolates obtained from clinical and environmental samples in a Greek ICU belonged to the Southeast clade I, being more related to the strain from India. Although all isolates were epidemiologically linked and possessed identical sequences, we cannot assume clonality of the isolates (i.e., belonging to a single strain), since the discriminatory power of MLST may not be sufficient to differentiate between isolates within the same clade. Moreover, *C. auris* from patient 1 was isolated from a non-COVID-19 patient and patient 2 has been transferred in the ICU from another hospital of Athens, suggesting different routes of transmission. Thus, a method with high discriminatory power (e.g., WGS) is required in order to investigate hospital transmission and the dissemination of isolates in Greek hospitals. In Europe, the first case of *C. auris* infection was imported from India in 2009, and most of *C. auris* isolates belong to the clade I, while a strain of the clade II was also found in Austria [43,44]. The first outbreak of *C. auris* in Europe occurred in a London cardio-thoracic center between April 2015 and July 2016 [7]. *C. auris* outbreaks have been recently reported in five European Region countries (Denmark, France, Germany, Greece and Italy), while at least two of them in Germany and Italy involved COVID-19 patients or units dedicated to the care of COVID-19 patients [45]. *C. auris* transmission showed interregional spread and regional endemicity in one country (Spain).

## 5. Conclusions

In conclusion, we demonstrated a case series of clade I *C. auris* isolations among patients for the first time in Greece. Although *C. auris* isolation might not always represent infection, it has the ability to survive in the hospital environment and enables the horizontal transmission of the organism. This was evident from the isolation of *C. auris* from environmental samples in the ICU. Thus, *C. auris* has been associated with hospital outbreaks, mainly in the ICU environment. Due to resistance against common disinfectants along with the multidrug resistance pattern to all classes of antifungals, *C. auris* is recognized as a serious concern for public health. Especially in the setting of the ongoing COVID-19 worldwide pandemic, a level of vigilance is warranted. Moreover, misidentification of *C. auris* when using common diagnostic platforms in routine can delay diagnosis. The current study has confirmed that *C. auris* is an emerging yeast pathogen in our region during the ongoing COVID-19 pandemic. Adherence to strict infection control is needed to restrain further spread of the pathogen.

## Figures and Tables

**Table 1:** Demographic and clinical characteristics of ICU patients with *C. auris*.

*C. auris* Cases	COVID-19 Status	Admission Date	Gender	Co-Morbidities	Age	Apache II Score	ICU Day at *C. auris* Isolation	LOS (Days)	ICU Outcome
Patient-1	Negative	19 October 2020	Male	paraplegia, diabetes mellitus	42	17	22	49	Death
Patient-2	Positive	1 August 2021	Female	metastatic adenocarcinoma of rectum (nephrostomy, ureterostomy)	57	23	9	10	Survival
Patient-3	Positive	10 August 2021	Female	diabetes mellitus, arterial hypertension, asthma	72	18	13	44	Survival
Patient-4	Positive	26 August 2021	Male	diabetes mellitus, arterial hypertension, painless jaundice (recently placed stent in the common bile)	78	12	9	37	Death
Patient-5	Positive	22 December 2021	Female	rheumatoid arthritis, renal resection (adenocarcinoma 6 years ago), Hashimoto thyroiditis	64	15	14	20	Death

**Table 2:** Antifungal susceptibility of *C. auris* clinical isolates.

*C. auris* Cases	Date of Isolation of	Source of Specimen	MIC (μg/mL) of Antifungal Agents							
			AMPHB ^1^	5-FLUC	FLU ^1^	VOR	POS	MICF	AND	ITR
Patient-1	30 November 2020	Deep hip decubitus Ulcer/urine	1	<0.0625	**>256**	>8	>8	0.12	0.12	2
Patient-2	23 August 2021	Urine	1	0.12	**>128**	>8	0.5	0.5	0.5	1
Patient-3	1 September 2021	Urine	**2**	<0.0625	**>128**	8	0.25	0.12	0.12	0.5
Patient-4	13 September 2021	Blood/ Urine	**2**	0.12	**>128**	0.5	0.25	0.12	0.12	0.25
Patient-5	13 January 2022	Bronchial aspirate	**2**	0.12	**>128**	0.12	0.12	0.25	0.25	0.06

AMPHB: AmphotericinB, 5-FLUC: 5-Flucytocine, FLU: Fluconazole, VOR: Voriconazole, POS: Posaconazole, MICF: Micafungin, AND: Anidulafungin, ITR: Itraconazole. ^1^ MIC values of resistant isolates are denoted in bold.

## Data Availability

The consensus nucleotide sequences of the ITS, D1/D2 RPB1, RPB2 and the genetic locus containing short tandem repeats (STRs) of the nonanucleotide TCCTTCTTC were deposited to GenBank database of NCBI and can be accessed online using their accession numbers (OP974692, OL455790, OL539420, OP981544and OP066795 respectively). All files related to the phylogenetic analysis of the studied *C. auris* isolates were uploaded to a custom Github repository (https://github.com/konskons11/Candida-auris, last accessed on 9 December 2022).

## Supplementary Materials

The following supporting information can be downloaded at: https://www.mdpi.com/article/10.3390/jof9020243/s1, Figure S1: MUSCLE (v3.8) alighnment of the CJJ09_001802 genetic locus containing short tandem repeats (STRs) of the nonanucleotide TCCTTCTTC.
