# Supplementary figures and images for: Emergence of Clonally-Related South Asian Clade I Clinical Isolates of Candida auris in a Greek COVID-19 Intensive Care Unit
Source: J Fungi (Basel). 2023 Feb 11;9(2):243. doi: 10.3390/jof9020243 (PMC9964037; doi:10.3390/jof9020243)

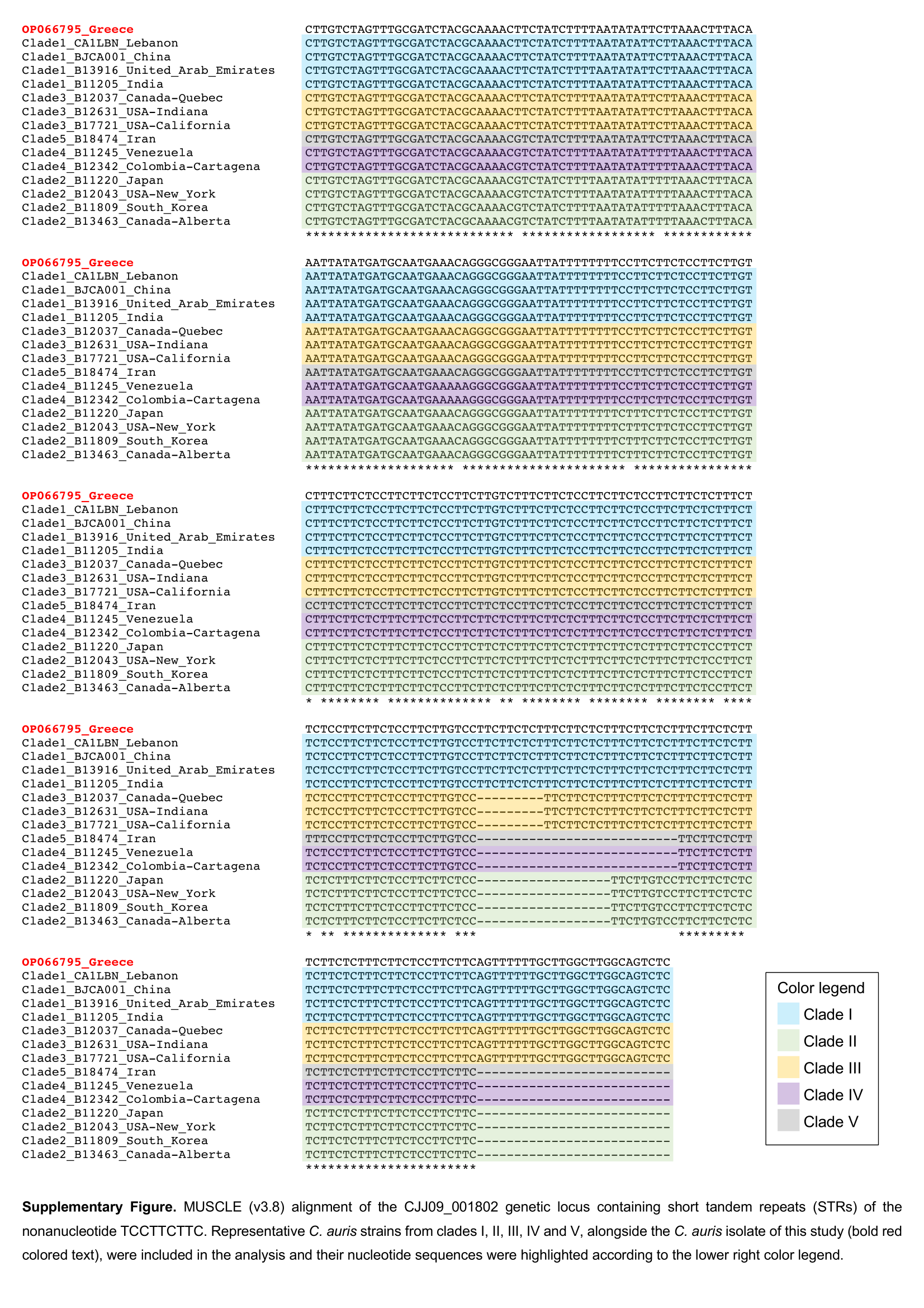

Supplement: Supplementary file 1 [file jof-09-00243-s001.zip › jof-2172435-supplementary.tiff]
